# Dysfunctional TLR1 reduces the therapeutic efficacy of chemotherapy by attenuating HMGB1-mediated antitumor immunity in locally advanced colorectal cancer

**DOI:** 10.1038/s41598-023-46254-1

**Published:** 2023-11-09

**Authors:** Kevin Chih-Yang Huang, Tao-Wei Ke, Jia-Yi Chen, Wei-Ze Hong, Shu-Fen Chiang, Chia-Ying Lai, Tsung-Wei Chen, Pei-Chen Yang, Liang-Chi Chen, Ji-An Liang, William Tzu-Liang Chen, K. S. Clifford Chao

**Affiliations:** 1 Department of Biomedical Imaging and Radiological Science, China Medical University, Taichung, 40402 Taiwan, ROC; 2 Translation Research Core, China Medical University Hospital, China Medical University, Taichung, 40402 Taiwan, ROC; 3 Cancer Biology and Precision Therapeutics Center, China Medical University, Taichung, 40402 Taiwan, ROC; 4 Department of Colorectal Surgery, China Medical University Hospital, China Medical University, Taichung, 40402 Taiwan, ROC; 5 School of Chinese Medicine and Graduate Institute of Chinese Medicine, China Medical University, Taichung, 40402 Taiwan, ROC; 6 Proton Therapy and Science Center, China Medical University Hospital, China Medical University, Taichung, 40402 Taiwan, ROC; 7 Lab of Precision Medicine, Feng-Yuan Hospital, Ministry of Health and Welfare, Taichung, 42055 Taiwan, ROC; 8 Department of Pathology, Asia University Hospital, Asia University, Taichung, 41354 Taiwan, ROC; 9 Department of Pathology, China Medical University Hospital, China Medical University, Taichung, 40402 Taiwan, ROC; 10 Department of Radiation Oncology, China Medical University Hospital, China Medical University, Taichung, Taiwan, ROC; 11 Department of Radiotherapy, School of Medicine, China Medical University, Taichung, 40402 Taiwan, ROC; 12 Department of Colorectal Surgery, China Medical University HsinChu Hospital, China Medical University, HsinChu, 302 Taiwan, ROC; 13 Department of Surgery, School of Medicine, China Medical University, Taichung, 40402 Taiwan, ROC

**Keywords:** Cancer, Gastrointestinal cancer, Tumour immunology

## Abstract

Regional lymph node metastasis is an important predictor for survival outcome and an indicator for postoperative adjuvant chemotherapy in patients with colorectal cancer. Even with advances in adjuvant chemotherapeutic regimens, 5-year distant metastasis and survival rates are still unsatisfactory. Here, we evaluate the clinical significance of polymorphisms in receptors for HMGB1, which is the hallmark of chemotherapy-induced immunogenic cell death, in patients with stage II–III colon carcinoma (COAD). We found that high cytosolic HMGB1 is elicited in stage III COAD patients who received adjuvant chemotherapy. Patients with the TLR1-N248S polymorphism (rs4833095), which causes loss-of-function in HMGB1-mediated TLR1–TLR2 signaling, may influence the therapeutic efficacy of adjuvant chemotherapy, leading to a high risk of distant metastasis within 5 years [HR = 1.694, 95% CI = 1.063–2.698, *p* = 0.027], suggesting that TLR1-N248S is an independent prognostic factor for locally advanced colon carcinoma patients. We found that defective TLR1 impaired TLR1/2 signaling during dendritic cell (DC) maturation for the antitumor immune response under immunogenic chemotherapy oxaliplatin (OXP) treatment. Defective TLR1 on DCs impaired their maturation ability by HMGB1 and reduced the secretion of IFNγ from T cells to eradicate tumor cells in vitro. Moreover, systemic inhibition of TLR1/2 dramatically reduced the tumor-infiltrating immune cells by OXP treatment, leading to poor therapeutic response to OXP. In contrast, administration of a TLR1/2 agonist synergistically increased the benefit of OXP treatment and triggered a high density of tumor-infiltrating immune cells. We also observed that fewer tumor-infiltrating cytotoxic T lymphocytes were located within the tumor microenvironment in patients bearing the TLR1-N248S polymorphism. Overall, our results suggest that dysfunctional TLR1 may reduce the therapeutic response to adjuvant chemotherapy by impairing HMGB1-mediated DC maturation and attenuating the antitumor immune response in locally advanced colon carcinoma patients.

## Introduction

Lymph node metastasis is a crucial predictor of survival outcome and an indicator for postoperative adjuvant chemotherapy in patients with colorectal cancer (CRC)^1–3^. Despite advances in therapeutic approaches such as combinational chemotherapy regimens, targeted therapy and radiotherapy, metastatic disease remains the leading factor in CRC mortality. Not all patients benefit from standard chemotherapy regimens, making it critical to stratify patients using predictive and prognostic biomarkers to identify those who will benefit the most from chemotherapy, such as immune-related genes^4^.

Toll-like receptors (TLRs) play a crucial role in intestinal mucosal innate and acquired immunity to maintain gut homeostasis^5^. TLRs not only recognize exogenous pathogen-associated molecular patterns (PAMPs) but also sensitize endogenous damage-associated molecular patterns (DAMPs) that are released from injured or dying cells^6^. These DAMPs shape adaptive anticancer immunity through the activation of antigen-presenting cells (APCs) for tumor antigen processing and cross-presentation to CD8^+^ T cells. Therefore, several groups have established prognostic biomarkers based on immunogenic cell death (ICD)-associated DAMP signatures to estimate prognosis and immunotherapy effectiveness in several malignancies, such as triple-negative breast cancer (TNBC) and head and neck squamous cell carcinoma (HNSCC)^7,8^. Several immunogenic chemotherapeutic drugs, such as oxaliplatin (OXP) and doxorubicin (DOX), promote ICD, releasing DAMPs, including high-mobility group box 1 (HMGB1), heat shock protein 70 (Hsp70), ATP, annexin A1 (ANXA1) and calreticulin (CRT)^9,10^. HMGB1 can activate TLR2 and TLR4 on dendritic cells (DCs) to initiate antigen presentation and cytokine secretion, activating antigen‐specific CD4^+^ T cells and CD8^+^ T cells to recognize and eradicate tumor cells. Furthermore, TLR2 agonists have significantly inhibited cancer growth by stimulating CD8^+^ T cells and natural killer (NK) cells^11,12^. TLR2 interacts with TLR1 or TLR6 as a functional heterodimer to activate downstream signaling in regulating the mucosal immune response within the gut^13^. Several single nucleotide polymorphisms (SNPs) in the TLR1 gene impair the response to several bacterial agonists for this receptor and affect T helper 1 cytokine production, including P315L (rs5743613), N248S (rs4833095) and I602I (rs5743618)^14^. These polymorphisms of TLR1 are associated with the response to FOLFIRI plus bevacizumab in metastatic CRC^15^. Additionally, the expression of TLR1 and TLR2 serves as prognostic factors in breast cancer and pancreatic ductal adenocarcinoma (PDAC)^16,17^. Recent studies have shown that the activation of TLR1/2 signaling enhances the antitumor efficacy of CTLA-4 blockade, suggesting that TLR1/2 signaling participates in not only innate immunity against bacterial infection but also adaptive immunity to eradicate tumor cells. Therefore, several TLR immunomodulators have been linked to anticancer therapies in preclinical studies and clinical trials^18,19^, including the TLR2 agonist SMU-Z1 and polysaccharide krestin (PSK)^11,20^, TLR3 agonist AMP‐516^21^, TLR5 agonist entolimod (CBLB502)^22^, TLR7 agonist imiquimod^23^ and TLR9 agonist CpG‐7909^24^.

In this study, we investigated the impact of genetic variation in TLR1 and TLR2, which are the receptors for HMGB1, on the clinical outcomes of colon carcinoma (COAD) patients who underwent postoperative adjuvant chemotherapy, specifically those with regional lymph node metastatic COAD. Our findings suggest that stage III COAD patients carrying the TLR1-N248 polymorphism exhibit poorer distant metastasis-free survival (DMFS) and disease-free survival (DFS). Defective TLR1 signaling decreases HMGB1-mediated dendritic cell (DC) maturation induced by immunogenic chemotherapy. Blocking TLR1/2 signaling significantly reduced the therapeutic efficacy of the immunogenic chemotherapeutic agent, resulting in reduced infiltration of DCs, CD4, and CD8 within the tumor microenvironment. However, administering a TLR1/2 agonist significantly increased the therapeutic efficacy of OXP by boosting the antitumor immune response. In conclusion, our results indicate that dysfunctional TLR1 reduces the extent of antitumor immunity elicited by immunogenic chemotherapy, leading to poor response to OXP and worse survival outcomes in locally advanced CRC patients.

## Material and methods

### Patient characteristics

From 2006 to 2014, 869 stage II–III colon carcinoma patients who were histologically and clinically diagnosed underwent surgery, followed by postoperative adjuvant chemotherapy (high-risk stage II and stage III patients), and were followed-up once every 6 months postoperatively for 5 years at China Medical University Hospital (CMUH). Adjuvant chemotherapy regimens were administered as previously described^5,14^. All study protocols were approved by the Institutional Review Board (IRB) of CMUH [Protocol number: CMUH105-REC2-073].

### Tissue microarray (TMA) construction and immunohistochemistry

Stage II–III colon carcinoma patients who were diagnosed and treated between 2006 and 2014 at China Medical University Hospital were enrolled in our cohort^25,26^. The TMA included resected primary tumor tissue and their corresponding normal mucosa specimens, which was approved by the Institutional Review Board (IRB) of China Medical University Hospital [Protocol number: CMUH105-REC2-073]. The collection of specimens and methods was carried out in accordance with the approval guidelines from the committee. Briefly, central areas of the resected primary tumor were marked on hematoxylin/eosin-stained slides by pathology, and the corresponding area on the matching formalin-fixed, paraffin-embedded tissue (FFPE) was then identified by punching 2-mm-diameter tissue cylinders. The criteria for TMA spots were at least 50% tumor cells^27^.

Immunohistochemistry (IHC) was performed using 3-μm-thick TMA sections with the indicated antibodies, followed by an HRP-conjugated avidin biotin complex (ABC) Kit (Vector Laboratories, CA, USA), incubation with HRP substrate DAB chromogen (Vector Laboratories) and counterstaining in hematoxylin^28,29^. The immune cell markers CD8 (ab217344, Abcam) were counted at 400× magnification under a microscope. The average number of TILs in five high-power fields (400× magnification) was scored by two pathologists. Cytosolic HMGB1 (ab18256, Abcam) expression was based on the H-score^25,30^, according to the intensity and percentage of positive cells for histoscore (H-score), which was calculated by a semiquantitative assessment of both the intensity of the staining (0: negative staining; 1: weak; 2: moderate; and 3: strong) and the percentage of immunopositive cells. The range of the H-score was from 0 to 300. The cytosolic HMGB1expression status was categorized as low or high according to the median value of the H-score.

### Genomic DNA extraction and SNP genotyping

Genomic DNA from nontumor tissues of rectal cancer patients was extracted from two 5-μm-thick FFPE slides using a QIAamp® DNA FFPE Extraction Kit (QIAGEN GmbH, Hilden, Germany). For SNP genotyping, 10 ng of total genomic DNA was used for PCR amplification and was performed using the iPLEX® HS panel on the MassARRAY® System (Agena Bioscience, San Diego, CA, USA), which employs matrix-assisted laser desorption/ionization time-of-fight mass spectrometry for amplicon detection (MALDITOF-MS; SpectroACQUIRE, Agena Bioscience). Primers designed (Supplementary Table S1) for PCR amplification of specific polymorphisms and extension reactions were prepared using MassARRAY® Assay Design Version 3.1 software (Agena Bioscience, San Diego, CA, USA). Following PCR, SAP addition, and the iPLEX HS® extension reaction, the samples were desalted by resin treatment for 15 min, spotted onto SpectroCHIP® Arrays (Agena Bioscience, San Diego, CA), analyzed by mass spectrometry, and ultimately interpreted using SpectroTYPER v4.0 software (Agena Bioscience, San Diego, CA). The characteristics of the selected polymorphisms are shown in Table S1.

### Cell line and reagents

Two human colorectal cancer cell lines, HT29 and HCT116, the monocytic leukemia cell line THP1 and human T-cell leukemia Jurkat cells, were cultured in RPMI 1640 medium supplemented with 10% fetal bovine serum (HyClone, MA, USA) at 37 °C with a humidified atmosphere of 5% CO_2_ and 95% air. CU-CPTT22 (TLR1/2 antagonist), TLR4-IN-C34 (TLR4 antagonist), FPS-ZM1 (RAGE antagonist) and Pam_3_CSK_4_ (TLR1/2 agonist) were purchased from MedChemExpress (MCE, NJ, USA).

### Western blot analysis

Total cell lysates (20–40 μg) were separated via 6–12% SDS‒PAGE and transferred onto a PVDF membrane (Millipore, MA, USA)^31^. The membranes were blocked with BlockPRO™ Blocking buffer (BioLion Biotech, Taipei, Taiwan), probed with specific antibodies overnight at 4 °C, and then incubated with HRP-conjugated secondary antibodies for 1 h. After antibody incubation, the membranes were incubated with Immobilon Western Chemiluminescent HRP Substrate (Millipore) and analyzed by an ImageQuant™ LAS 4000 biomolecular imager (GE Healthcare, Amersham, UK). The digital data were processed using Adobe Photoshop and quantified using ImageJ software (NIH, MD, USA). Each blot was stripped by immunoblotting stripping buffer (BioLion Tech.) before reprobing with the other antibodies. The antibodies used in western blot analysis were as follows: anti-p-IKKα/β (AP0546, Abclonal), anti-p-JNK (AP0631, Abclonal) and anti-TLR1 (A0997, Abclonal)^28,29^.

### THP1-derived immature DCs and colorectal cancer cell line coculture

The human monocytic leukemia cell line THP1 was cultured and maintained in RPMI 1640 medium supplemented with 10% FCS (Life Technologies, Grand Island, New York, USA), 2 mM glutamine, 1 mM sodium pyruvate, 100 U/ml penicillin, and 100 mg/ml streptomycin at 37 °C in a humidified incubator with 95% air and 5% CO_2_. After the gene was silenced by lentivirus carrying shRNA against the indicated genes, immature DCs were generated from THP1 cells as previously described^32^. Briefly, THP1 cells were differentiated into immature DCs by adding 1500 IU/ml rhIL-4 (Sino Biological, Beijing, China) and 1500 IU/ml rhGM-CSF (Sino Biological, Beijing, China) in culture medium for at least 7 days, with cytokine-supplemented culture medium changed every 2–3 days at 37 °C in a humidified incubator with 95% air and 5% CO_2_. These THP1-iDC cells were treated with recombinant human HMGB1 (Sino Biological, Beijing, China) for 24 h. DC maturation was measured by flow cytometry. These THP1-iDC cells were also cocultured with OXP-treated HT29-GFP cells to analyze the DC maturation marker CD86 by flow cytometry. In addition, the human T-cell leukemia Jurkat cell line was coincubated with OXP-treated HT29/THP1-iDCs for 15 h to analyze the level of IFNγ.

### Animal model

BALB/c mice (female, 5 weeks old) were maintained according to the institutional guidelines approved by the Institutional Animal Care and Use Committee of China Medical University [Protocol number: CMUIACUC-2020-057]. CT26 cells (2 × 10^5^ cells/mouse) were suspended in 100 μL 20% Matrigel and subcutaneously inoculated into the right flank of each mouse. After 10 days, mice were administered oxaliplatin (OXP, 6 mg/kg/mouse, intraperitoneal injection) 4 times at 3-day intervals on Days 13, 16, 19, 22 and 25. TLR1/2 antagonist CU-CPT22 (2.5 mg/kg, HY-108471, MCE) and TLR1/2 agonist Pam_3_CSK_4_ (2.5 mg/kg, HY-P1180, MCE). The tumor volume was measured at 3-day intervals in the study, and tumor volumes were calculated according to the formula (width^2^ × length)/2. All animals were sacrificed appropriately at the end of the experiments with CO_2_, and the tumor tissues were collected for further analysis, including immunoblotting analysis and immunohistochemistry^33,34^. The study was performed with the protocols in the Institutional Animal Care and Use Committee of China Medical University and carried out in compliance with the ARRIVE guidelines^35^.

### Tumor processing to isolate tumor-infiltrating lymphocytes (TILs) and lymph nodes for flow cytometry

For analysis of tumor-infiltrating immune cells, mice from each treatment group were sacrificed on Day 28, and their tumors and tumor-draining lymph nodes were isolated. Isolated tumors were weighed, and mechanically dissected into small pieces (1–2 mm) by a beaver blade, filtered through a 70-μm nylon cell strainer, and then resuspended in blank RPMI medium. Thereafter, the cell suspensions were layered over Ficoll-Paque media and centrifuged at 1025×*g* for 20 min. The layer of mononuclear cells was transferred into a conical tube, 20 ml of complete RPMI media was added, and then the cells were gently mixed and centrifuged at 650×*g* for 10 min twice. Finally, the supernatant was removed, and the TILs were resuspended in complete RPMI medium^36^.

For Treg staining, these cells were fixed and permeabilized with a FoxP3 Fix/Perm buffer kit from Biolegend according to the manufacturer’s instructions and then stained with intracellular antibodies for further analysis by flow cytometry. For quantification, the absolute numbers of different cell types per gram of tumor were measured by flow cytometric analysis. For surface marker staining, TILs were resuspended in 500 μL of staining buffer (2% BSA, 0.1% NaN_3_ in PBS). The cells were stained with different surface marker panels: (1) T cells phenotype: CD3-FITC (100,204, Biolegend, CA, USA), CD4-APC/Fire^TM^750 (116,020, Biolegend, CA, USA), CD8a-PerCPCy5.5 (100,734, Biolegend, CA, USA), CD44-PE (103,008, Biolegend, CA, USA), CD45-PECy7 (103,114, Biolegend, CA, USA), CD62L-APC (104,412, Biolegend, CA, USA) and their isotype; (2) Regulatory T lymphocyte (Treg): FoxP3-Alexa488 (126,406, Biolegend, CA, USA), CD25-PE (101,904, Biolegend, CA, USA), CD3-PerCPCy5.5 (100,218, Biolegend, CA, USA), CD45-PECy7 (103,114, Biolegend, CA, USA), CD127-Alexa647 (135,020, Biolegend, CA, USA), CD4-APC/Fire^TM^750 (116,020, Biolegend, CA, USA), and their isotype; (3) IFNγ^+^CD8^+^ T cells: CD3-FITC (100,204, Biolegend, CA, USA), IFNγ-PE (505,808, Biolegend, CA, USA), CD45-PECy7 (103,114, Biolegend, CA, USA), CD8-APC/Fire^TM^750 (100,766, Biolegend, CA, USA) and their isotype. Data were acquired on a Guava easyCyte flow cytometer and analyzed by FlowJo Software^37,38^.

### Statistical analysis

The statistical analysis was performed by SPSS (IBM SPSS Statistics 22, WA, USA), and GraphPad Prism 7 (GraphPad Software, San Diego, CA, USA) was utilized. A two-sided *p* < 0.05 was used as the significance level for all tests, such as Student’s *t* test, Pearson chi-square test and Fisher’s exact test. Univariate and multivariate analyses were performed by Cox regression analysis to estimate the hazard ratios (HRs) and 95% confidence intervals (CIs). These influential factors were adjusted in the Cox models, including sex (male vs. female), age (≥ 65 vs. < 65), pT stage (pT 3–4 vs. pT 1–2), tumor location (proximal vs. distal), tumor differentiation (poor vs. well to moderate), LVI (present vs. absent), PNI (present vs. absent) and TLR1-N248 (variant vs. WT) and TLR2 promoter (variant vs. WT). Kaplan‒Meier analysis was used to assess distant metastasis-free survival (DMFS) and disease-free survival (DFS) with the survival time between surgery and events such as tumor relapse and death.

### Ethical approval

This study was reviewed and approved by the Internal Review Board (IRB) of China Medical University Hospital [Protocol number: CMUH105-REC2-073]. The method was carried out in accordance with the committee’s approved guidelines.

### Informed consent

Informed consents were obtained from all participants in the study.

## Results

### Clinical characteristics and genotype of TLR1/TLR2, which are HMGB1 receptors, in stage II–III patients

**Figure 1:**
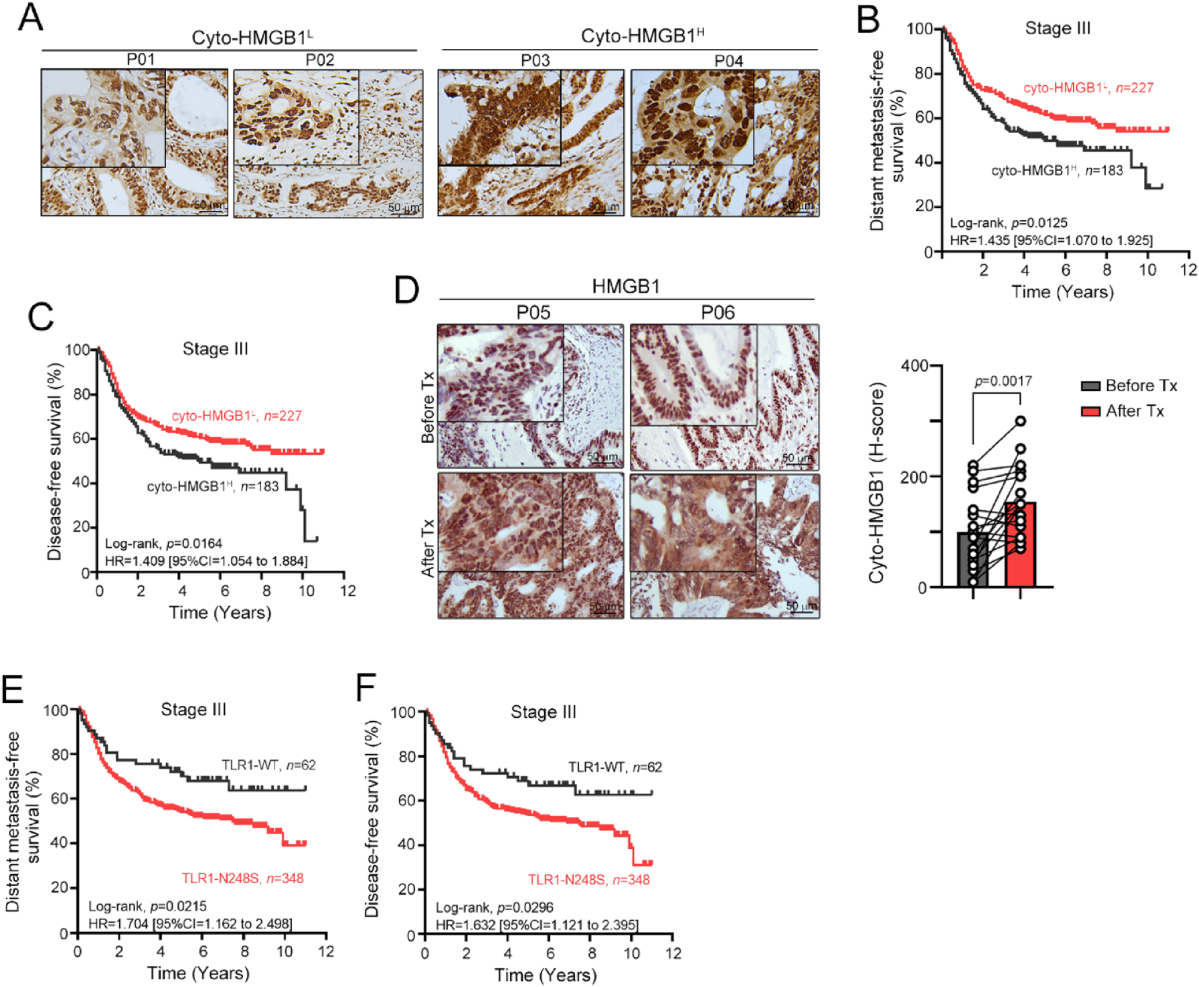
The association between distant metastasis-free survival (DMFS), disease-free survival (DFS) and TLR1 genotypes in locally advanced stage III COAD patients. (**A**) Representative images of cyto-HMGB1 expression within the tumor microenvironment in advanced colorectal cancers. (**B**) Kaplan–Meier curves showed that inflammation-driven cyto-HMGB1 expression was associated with poor DMFS in locally advanced COAD patients (*n* = 410, *p* = 0.0125). (**C**) Kaplan–Meier curves showed that inflammation-driven cyto-HMGB1 expression was associated with poor DFS in locally advanced COAD patients (*n* = 410, *p* = 0.0164). (**D**) The intensity of cyto-HMGB1 (H-score) within the TME in colorectal cancer patients before and after chemotherapy treatment was evaluated by IHC (*n* = 19, *p* = 0.0017, unpaired t test). (**E**) Kaplan–Meier curves showed that TLR1-WT (AA genotype) is associated with favorable DMFS in locally advanced COAD patients compared to TLR1-N248S (AG and GG genotypes). (**F**) Kaplan–Meier curves showed that TLR1-WT (AA genotype) is associated with favorable DFS in locally advanced COAD patients compared to TLR1-N248S (AG and GG genotypes).

Our previous studies have demonstrated that the density and cytosolic pattern of HMGB1 (cyto-HMGB1) is an independent prognostic factor in locally advanced rectal cancer patients who underwent preoperative concurrent chemoradiotherapy, suggesting that chemoradiotherapy elicits the release of HMGB1 to promote ICD and antitumor immunity^26^. In this study, we investigated whether cyto-HMGB1 is associated with survival outcomes in patients with locally advanced colon adenocarcinoma (COAD) who received adjuvant chemotherapy. As shown in Fig. 1A–C, high cyto-HMGB1 levels in tumor tissues of stage III COAD patients were associated with poor distant metastasis-free survival (DMFS) and disease-free survival (DFS). Patients with low cyto-HMGB1 had a negative correlation with higher nuclear HMGB1 expression and were associated with a more favorable survival outcome, which was consistent with previous studies^39^. These results suggest that environment-driven inflammation for cyto-HMGB1 before chemotherapy may promote tumor progression and inhibit antitumor immunity with other mechanisms, such as autophagy^39–43^. However, chemotherapy-induced ICD signatures may be associated with antitumor immunity and influence the risk of distant metastasis and the therapeutic efficacy of chemotherapy^8^. Our results demonstrated that cyto-HMGB1 was significantly increased in advanced-stage CRC patients after chemotherapy treatment (Fig. 1D, p = 0.0017).

To evaluate whether genetic defects in TLR1, TLR2 and TLR4 may influence HMGB1-mediated antitumor immunity and be associated with distant metastasis and tumor relapse in advanced-stage CRC patients after chemotherapy treatment. We evaluated the genetic polymorphisms of TLR1/2, namely, TLR1 (N248S/rs4833095), TLR1 (S602I/rs5743618), TLR2 (N729S/rs61735278), TLR2 promoter (− 196 to − 174 deletion/rs111200466), TLR4 (D299G/rs4986790) and TLR4 (T399I/rs4986791), in stage II–III COAD patients (n = 869). The individual genotypes and allele frequencies were shown and were consistent with the global allele frequencies in Table S1, which were classified based on the NCBI dbSNP. The genotype GG of TLR1 (N248S/rs4833095) and promoter deletion of TLR2 (− 196 to − 174 deletion/rs111200466) were prevalent among COAD patients (Table 1, *n* = 869). The mean age at diagnosis was 65.14 ± 13.65 years (range, 13–92 years). The majority of the patients were men (53.9%). After surgery, 60 patients (6.9%) exhibited local recurrence, and 147 patients (16.9%) exhibited distant metastasis within 10 years.

**Table 1:** Clinicopathological parameters of stage II–III colon carcinoma patients (n = 869).

Clinicopathological parameters	Total no	TLR1-N248		*p* value	TLR1-S602		*p* value	TLR2-N729S		*p* value	TLR2 promoter (− 196 to − 174)		*p* value
		WT	Variant		WT	Variant		WT	Variant		WT	Variant	
	869	144	725		853	16		867	2		373	494	
Gender				0.516			0.846			1			0.416
Female	401	70	331		394	7		400	1		178	222	
Male	468	74	394		459	9		467	1		195	272	
Age				0.075			0.277			0.199			0.369
< 65	388	74	314		383	5		386	2		173	214	
≥ 65	481	70	411		470	11		481	0		200	280	
Tumor location				0.272			0.127			1			0.421
Proximal colon	398	59	339		394	4		397	1		165	233	
Distant colon	466	82	384		454	12		465	1		205	259	
Unspecified	5	3	2		5	0		5	0		3	2	
pT stage				0.821			1			1			0.134
pT1-2	33	5	28		33	0		33	0		10	23	
pT3-4	835	139	696		819	16		833	2		362	471	
pN stage				0.278			0.198			1			0.579
Negative	459	82	377		448	11		458	1		193	265	
Positive	410	62	348		405	5		409	1		180	229	
TNM stage (7th AJCC)				0.278			0.198			1			0.579
Stage II	459	82	377		448	11		458	1		193	265	
Stage III	410	62	348		405	5		409	1		180	229	
Tumor differentiation				1.00			1.00			1.00			0.25
Well to moderate	850	140	710		835	15		848	2		365	483	
Poor	7	1	6		7	0		7	0		1	6	
Unknown	12	3	9		11	1		12	0		7	5	
MMR status				0.865			1.00			1.00			0.312
pMMR	796	131	665		781	15		794	2		339	455	
dMMR	69	12	57		68	1		69	0		34	35	
Unknown	4	1	3		4	0		4	0		0	4	
Lymphovascular invasion				0.998			0.153			1			0.85
Absent	423	70	353		418	5		422	1		183	239	
Present	441	73	368		430	11		440	1		188	252	
Unknown	5	1	4		5	0		5	0		2	3	
Perineural invasion				0.532			0.621			0.529			0.093
Absent	535	85	451		527	9		534	2		242	293	
Present	326	57	269		319	7		326	0		128	197	
Unknown	8	2	6		8	0		8	0		4	4	
Local recurrence				0.734			0.619			1			0.116
Absent	809	135	674		793	16		807	2		353	454	
Present	60	9	51		60	0		60	0		20	40	
Distant metastasis				0.93			1			0.31			0.748
Absent	722	140	602		708	14		721	1		308	412	
Present	147	24	123		145	2		146	1		65	82	
Adjuvant chemotherapy				0.553			0.435			0.14			0.996
Yes	325	57	268		321	4		323	2		139	184	
No	544	87	457		532	12		544	0		234	310	

Pearson test was used, and Fisher’s exact test was used when counts were less than 5. The test did not include the “unknown or unspecified” group.

### Association of SNPs with clinical outcome in stage II–III COAD patients

Because the prevalence of TLR1 (S602I/rs5743618), TLR2 (N729S/rs61735278) and TLR4 (D299G/rs4986790 and T399I/rs4986791) is lower, we only evaluated the relationship of TLR1-N248S and TLR2 promoter (− 196 to − 174 deletion) to DMFS and DFS. As shown in Table 2, older age (59% vs. 80%, *p* < 0.001) and presence of lymphovascular invasion (LVI, 61% vs. 71%, *p* = 0.01) exhibited a significantly higher risk in DMFS and DFS, and presence of perineural invasion (PNI, 59% vs. 70%, *p* = 0.033) was significantly associated with poor 5-year DFS in stage II COAD patients by Kaplan–Meier survival analysis. The presence of TLR1-N248S and the TLR2 promoter (− 196 to − 174 deletion) was not associated with DMFS and DFS in stage II COAD patients. However, the variants of TLR1-N248 (AG/GG genotype) had a notably higher risk in DMFS (52% vs. 68%, *p* = 0.019) and DFS (51% vs. 66%, *p* = 0.026) in stage III COAD, suggesting that the TLR1-N248S polymorphism may be associated with regional lymph node metastasis.

**Table 2:** Correlation between clinicopathologic parameters, DMFS and DFS.

Parameters	No^a^	Stage II				No^a^	Stage III			
		DMFS (%)	*p* value*	DFS (%)	*p* value*		DMFS (%)	*p* value*	DFS (%)	*p* value*
	459	68		66		410	54		53	
Sex			0.133		0.107			0.945		0.986
Female	212	72		71		189	54		53	
Male	247	64		63		221	55		53	
Age			< 0.001		< 0.001			< 0.001		< 0.001
**< **65	194	80		79		194	66		65	
≥ 65	265	59		57		216	44		43	
pT stage			NA		NA			< 0.001		< 0.001
pT1-2	1	0		0		32	88		88	
pT3-4	458	68		67		377	52		50	
Tumor location			0.454		0.583			0.052		0.085
Distal colon	245	69		68		221	58		57	
Proximal colon	212	66		65		186	50		50	
Tumor differentiation			0.214		0.232			0.67		0.688
Well to moderate	448	68		67		402	54		53	
Poor	5	40		40		2	50		50	
Lymphovascular invasion			0.010		0.005			0.039		0.021
Absent	309	71		70		114	62		62	
Present	147	61		59		294	51		50	
Perineural invasion			0.055		0.033			0.008		0.002
Absent	326	71		70		210	61		61	
Present	128	62		59		198	48		46	
TLR1-N248			0.34		0.36			0.019		0.026
WT	82	62		61		62	68		66	
Variant	377	69		68		348	52		51	
TLR2 promoter (− 196 to − 174)			0.747		0.806			0.732		0.503
WT	265	66		66		229	53		51	
Variant	193	69		67		180	56		56	

^a^Number of cases may differ due to missing data.

* indicated *p* < 0.05.

In the univariate analysis of DMFS in locally advanced stage III COAD patients, the following parameters were associated with patient survival rate: age, pT stage, LVI and PNI. Moreover, patients with the TLR1-N248 polymorphism had an increased risk for lower DMFS (HR = 1.725, 95% CI = 1.084–2.745, *p* = 0.0215, Fig. 1E) and DFS (HR = 1.632, 95% CI = 1.121–2.395, *p* = 0.0296, Fig. 1F) compared with patients carrying wild-type TLR1 (Table 3). These results indicate that the TLR1-N248 polymorphism has significant prognostic value for locally advanced stage III COAD patients. Subsequently, we examined whether the inclusion of other variables was significantly associated with the survival of locally advanced COAD. Our results showed that the TLR1-N248 polymorphism (HR = 1.694, 95% CI = 1.063–2.698, *p* = 0.027) was an independent prognostic factor of DMFS for locally advanced COAD patients (Table 3), indicating that the TLR1-N248 polymorphism has significant prognostic value for COAD patients with regional lymph node metastasis. These results suggested that loss-of-function in TLR1 may impair the immunogenic effect of released HMGB1 on antitumor immunity elicited by immunogenic chemotherapeutic agents such as oxaliplatin (OXP).

**Table 3:** Univariate and multivariate analysis of DMFS and known prognostic factors in stage III colon carcinoma patients.

Parameters	Univariate analysis			Multivariate analysis		
	HR	95% CI	*p* value	HR	95% CI	*p* value
Sex (Male vs. Female)	0.99	0.743–1.320	0.946			
Age (≥ 65 vs. < 65)	1.86	1.377–2.510	< 0.001	1.826	1.35–2.469	< 0.001
pT stage (pT3-4 vs. pT1-2)	5.278	1.959–14.220	0.001	4.489	1.653–12.190	0.003
Tumor location (Proximal vs. Distal)	0.753	0.565–1.005	0.054			
Tumor differentiation (Poor vs. Well to moderate)	1.523	0.213–10.879	0.675			
Lymphovascular invasion (Present vs. Absent)	1.428	1.014–2.009	0.041	1.26	0.889–1.784	0.194
Perineural invasion (Present vs. Absent)	1.476	1.103–1.974	0.009	1.224	0.909–16.49	0.183
TLR1-N248 (Variant vs. WT)	1.725	1.084–2.745	0.021	1.694	1.063–2.698	0.027
TLR2 promoter (Variant vs. WT)	1.052	0.787–1.406	0.734			

### Defective TLR1 signaling attenuated OXP-elicited TLR1/TLR2 signaling activation

**Figure 2:**
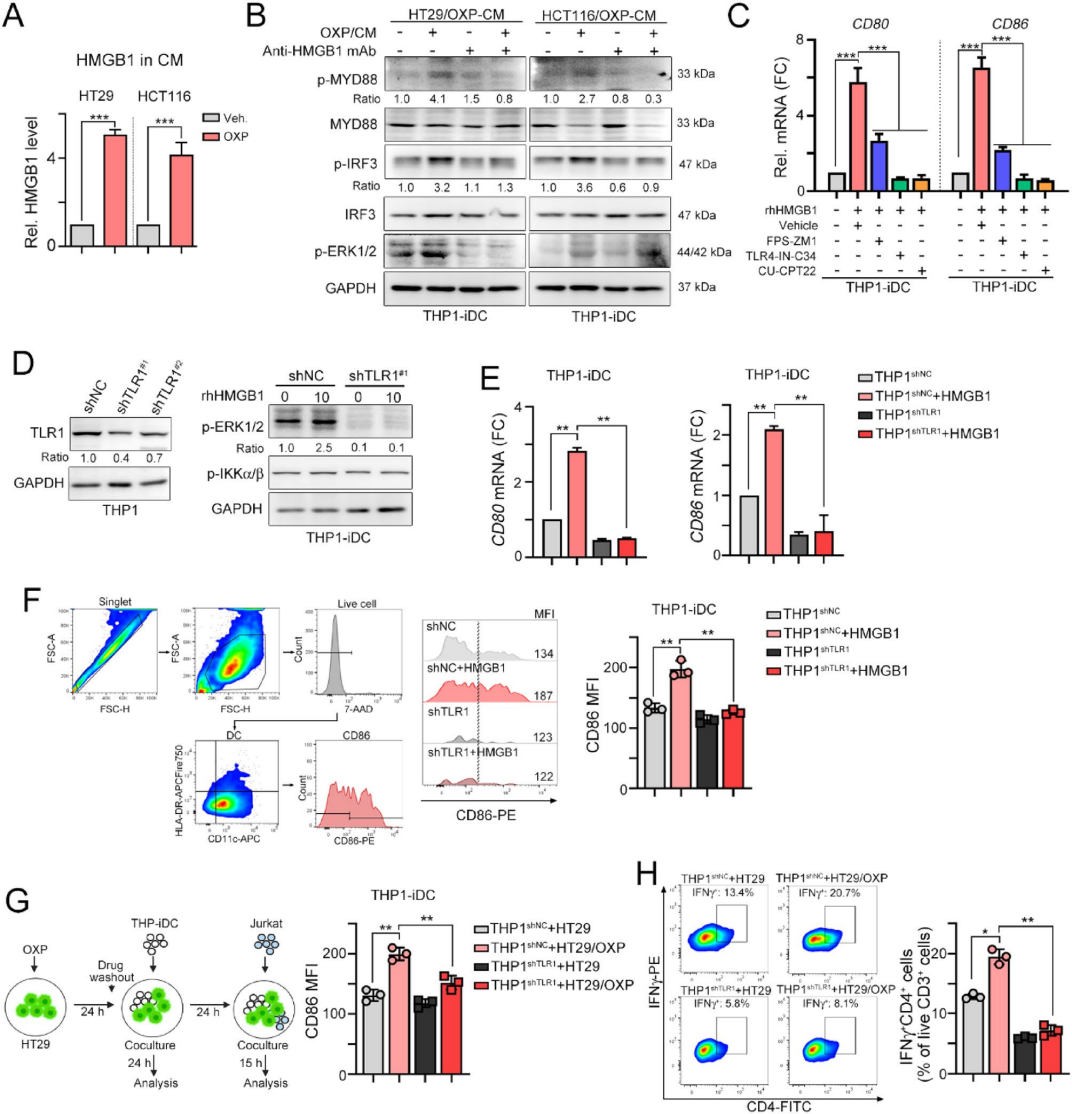
Knockdown of TLR1 reduced TLR1/2 signaling for DC maturation. (**A**) The level of HMGB1 in conditioned medium (OXP: 25 μM) after 24 h treatment was evaluated by an ELISA kit (*n* = 3). One-way ANOVA t test, ***p < 0.001. (**B**) The conditioned medium from OXP (25 μM)-treated HT29 and HCT116 cells was collected after 24 h of treatment. These CMs were preincubated with anti-HMGB1 antibodies (1 μg/mL) for 0.5 h and then added to THP1-iDCs, which were differentiated into immature DCs (iDCs) by IL-4 (1500 IU/ml) and GM-CSF (1500 IU/ml) for 7 days. (**C**) THP1-iDCs were treated with different small molecules for 1 h, and then rhHMGB1 was added for 24 h to analyze the maturation of DCs (*CD86* and *CD80*) by qRT‒PCR (*n* = 3). One-way ANOVA t test, **p < 0.01. (**D**) TLR1 was knocked down in THP1 cells by lentivirus carrying shRNA with a spinoculation protocol. Then, THP1-iDCs were treated with rhHMGB1 for 24 h for analysis by immunoblotting. (**E**) THP1^shNC^-iDCs and THP1^shTLR1^-iDCs were treated with rhHMGB1 for 24 h to analyze the maturation of DCs (*CD86* and *CD80*) by qRT‒PCR (*n* = 3). One-way ANOVA t test, **p < 0.01. (**F**) THP1^shNC^-iDCs and THP1^shTLR1^-iDCs were treated with rhHMGB1 protein for 24 h. DC maturation CD86 was analyzed by flow cytometry (*n* = 3). One-way ANOVA t test, **p < 0.01. (**G**) The diagram scheme of coculture analysis. HT29-GFP cells were treated with OXP (20 μM) for 24 h, and then the drugs were washed to coculture with THP1^shNC^-iDCs and THP1^shTLR1^-iDCs for 24 h. DC maturation of CD86 was analyzed by flow cytometry (*n* = 3). One-way ANOVA t test, **p < 0.01. (**H**) HT29 cells were treated with OXP (20 μM) for 24 h, and then the drugs were washed out and cocultured with THP1^shNC^-iDCs and THP1^shTLR1^-iDCs for 24 h. After coculture with iDCs for 24 h, the Jurkat T cells were coincubated for 15 h to analyze the IFNγ^+^ T cells by flow cytometry (*n* = 3). One-way ANOVA t test, *p < 0.05 and **p < 0.01.

To demonstrate that defective TLR1 impairs HMGB1-mediated TLR1/2 signaling for DC maturation by immunogenic chemotherapy, we first treated HT29 and HCT116 cells with OXP and analyzed the level of released HMGB1 (Fig. 2A). Indeed, we found that released HMGB1 was clearly detected in conditioned medium (CM) after OXP treatment (Fig. 2A). These OXP-treated CM from HT29 and HCT116 cells significantly triggered the activation of the TLR-mediated downstream, MYD88, ERK and IRF3 signaling pathway in THP1-iDC cells (Fig. 2B). However, the addition of anti-HMGB1 neutralizing antibodies clearly diminished the activation of the TLR-mediated signaling pathway, suggesting that HMGB1 released by OXP was critical to enhance DC maturation by TLR receptors. The released HMGB1 promoting DC maturation was mainly dependent on TLR2, TLR4 and RAGE for antitumor immunity^26,44,45^. Therefore, we combined several small molecule inhibitors, including FPS-ZM1 (RAGE antagonist), TLR4-IN-C34 (TLR4 antagonist) and CU-CPTT22 (TLR1/2 antagonist), with HMGB1 recombinant protein (rhHMGB1) to evaluate DC maturation. We found that blockade of TLR1/2, TLR4 and RAGE significantly decreased HMGB1-enhanced DC maturation (Fig. 2C). The level of DC maturation markers *CD80* and *CD86* were significantly decreased (Fig. 2C). But we found that TLR1/2 and TLR4 mainly participate in HMGB1-mediated DC maturation, compared to RAGE signaling pathway.

We assume that dysfunctional TLR1 may attenuate HMGB1-activated TLR1/TLR2 signaling for DC maturation by OXP to influence its therapeutic efficacy. Therefore, we generated TLR1-silenced THP1 monocytes (Fig. 2D) and differentiated them into immature DCs (THP1-iDCs) by the cytokines IL4 and GM-CSF. These THP1-iDC cells were treated with rhHMGB1 protein to analyze the downstream effects of TLR1/2 signaling (Fig. 2D). We found that treatment with HMGB1 markedly activated TLR1/TLR2 signaling. However, silencing TLR1 led to a significant impact on the activation of TLR1/2 signaling in THP1-iDC cells, especially ERK activation (Fig. 2D). Additionally, the DC maturation markers *CD80* and *CD86* were significantly decreased (Fig. 2E). The surface DC maturation marker CD86 was also decreased in THP1^shTLR1^-iDC cells, suggesting that defective TLR1 directly impacts TLR1/2 signaling for DC maturation (Fig. 2F).

We then cocultured OXP-treated HT29-GFP cancer cells with THP1^shNC^-iDCs and THP1^shTLR1^-iDCs for 24 h (Fig. 2G). We found that the DC maturation marker CD86 was significantly decreased in THP1^shTLR1^-iDC cells after coculture with OXP-treated HT29-GFP cells compared to THP1^shNC^-iDC cells (Fig. 2G). Then, we cocultured Jurkat T lymphocytes with OXP-treated HT29/THP1-iDCs to evaluate the level of the cytotoxic marker IFNγ (Fig. 2H). As shown in Fig. 2H, we found that the level of IFNγ in Jurkat T lymphocytes was also significantly decreased in THP1^shTLR1^-iDCs (Fig. 2H). These results suggested that defective TLR1 attenuated TLR2 signaling, leading to less DC maturation and T-cell activation by immunogenic chemotherapy.

### TLR1/2 signaling influences the therapeutic efficacy of chemotherapy by boosting antitumor immunity

**Figure 3:**
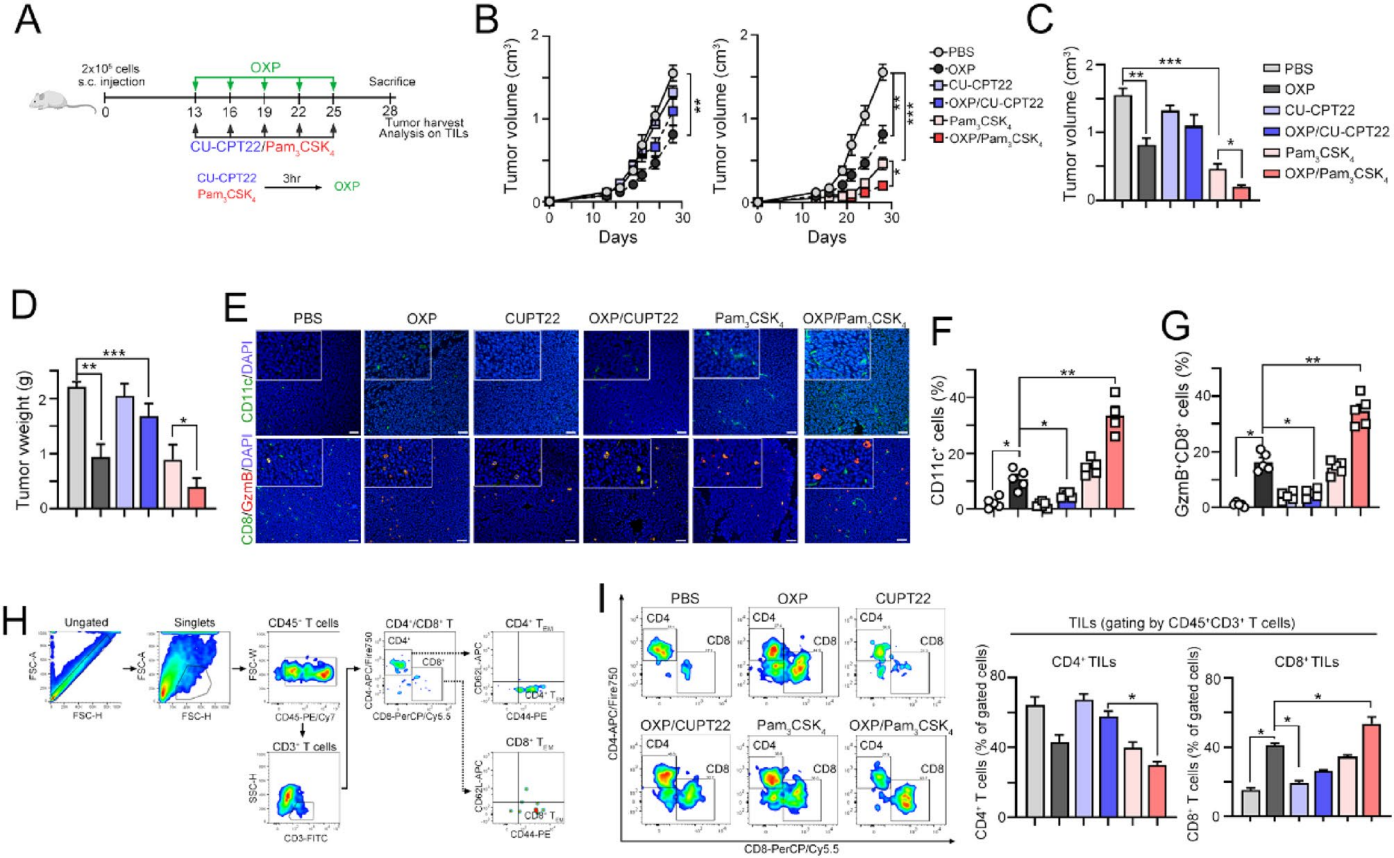
Systemic inhibition of TLR1/2 signaling reduced the therapeutic efficacy of chemotherapy in vivo. (**A**) Diagram of the animal experiment. (**B**) CT26 cells were subcutaneously inoculated into BALB/c mice for 13 days as the tumor volume reached 100 mm^3^. After randomization into 6 groups, OXP (6 mg/kg/mouse, *i.p.*) was administered on Days 13, 16, 19, 22 and 25. The TLR1/2 antagonist CU-CPT22 (2.5 mg/kg/mouse) and TLR1/2 agonist Pam_3_CSK_4_ (2.5 mg/kg/mouse) were administered 3 h before OXP administration. Tumor volume was measured every 3 days (*n* = 5–6). Two-way ANOVA t test, *p < 0.05, **p < 0.01 and ***p < 0.001. (**C**) The tumor volume was recorded on the end-point day (Day 28, *n* = 5–6). One-way ANOVA t test, *p < 0.05, **p < 0.01 and ***p < 0.001. (**D**) The resected tumor weight was recorded (*n* = 5–6). One-way ANOVA t test, *p < 0.05, **p < 0.01 and ***p < 0.001. (**E**) The densities of CD11c^+^ DCs and CD8^+^ T cells in resected tumors were analyzed by immunohistochemistry (*n* = 3–4). (**F**) The quantification of CD11c^+^ DCs in resected tumors is shown (*n* = 5). One-way ANOVA t test, *p < 0.05 and **p < 0.01. (**G**) The quantification of GzmB^+^CD8^+^ T cells in resected tumors is shown (*n* = 5). One-way ANOVA t test, *p < 0.05 and **p < 0.01. (**H**) Gating strategy of the tumor-infiltrating immune cell profile. (**I**) The phenotype of CD3^+^CD45^+^ tumor-infiltrating immune cells was analyzed by flow cytometry (*n* = 4–5). The percentage of CD4^+^ T cells (CD4^+^CD3^+^CD45^+^ T cells) and CD8^+^ T cells (CD8^+^CD3^+^CD45^+^ T cells) in total T cells (CD3^+^CD45^+^ T cells) was examined. One-way ANOVA t test, *p < 0.05.

To further validate that the therapeutic efficacy of chemotherapy was influenced by TLR1/2 signaling via antitumor immunity, we combined either the TLR1/2 antagonist CU-CPT22 or the TLR1/2 agonist Pam_3_CSK_4_ with OXP (Fig. 3A). Mice were given a subcutaneous tumor challenge with CT26 for 13 days and treated with OXP with or without CU-CPT22 and Pam_3_CSK_4_. We found that tumor volume was decreased in mice receiving 5 cycles of OXP (Fig. 3B). By pharmacologic inhibition of TLR1/2 signaling, we found that the therapeutic efficacy of OXP was significantly inhibited by administration of CU-CPT22 (Fig. 3B–D). In contrast, tumor regression was markedly reduced in the OXP/Pam_3_CSK_4_ group compared to mice that received OXP only (Fig. 3B–D), suggesting that activation of TLR1/2 signaling may enhance the therapeutic efficacy of OXP.

We then evaluated the immune cell profile within the tumor microenvironment (TME) by flow cytometry and IHC. The density of tumor-infiltrating CD11c^+^ DCs and GzmB^+^CD8^+^ T cells was markedly decreased in the OXP/CU-CPT22 group (Fig. 3E–G). However, there was an increase in the OXP/Pam_3_CSK_4_ group compared to the OXP group (Fig. 3E–G). By flow cytometric analysis, we found that tumor-infiltrating CD8^+^ T cells were also significantly increased in the OXP/Pam_3_CSK_4_ group compared to the other groups (Fig. 3H,I). Blockade of TLR1/2 significantly reduced the infiltration of CD8 T cells elicited by OXP treatment (Fig. 3H,I), suggesting that activation of TLR1/2 signaling may enhance the therapeutic efficacy of OXP by boosting antitumor immunity. But we found that the density of tumor-infiltrating CD4^+^ T cells were significantly decreased in the OXP/Pam_3_CSK_4_ group compared to the other groups (Fig. 3I).

**Figure 4:**
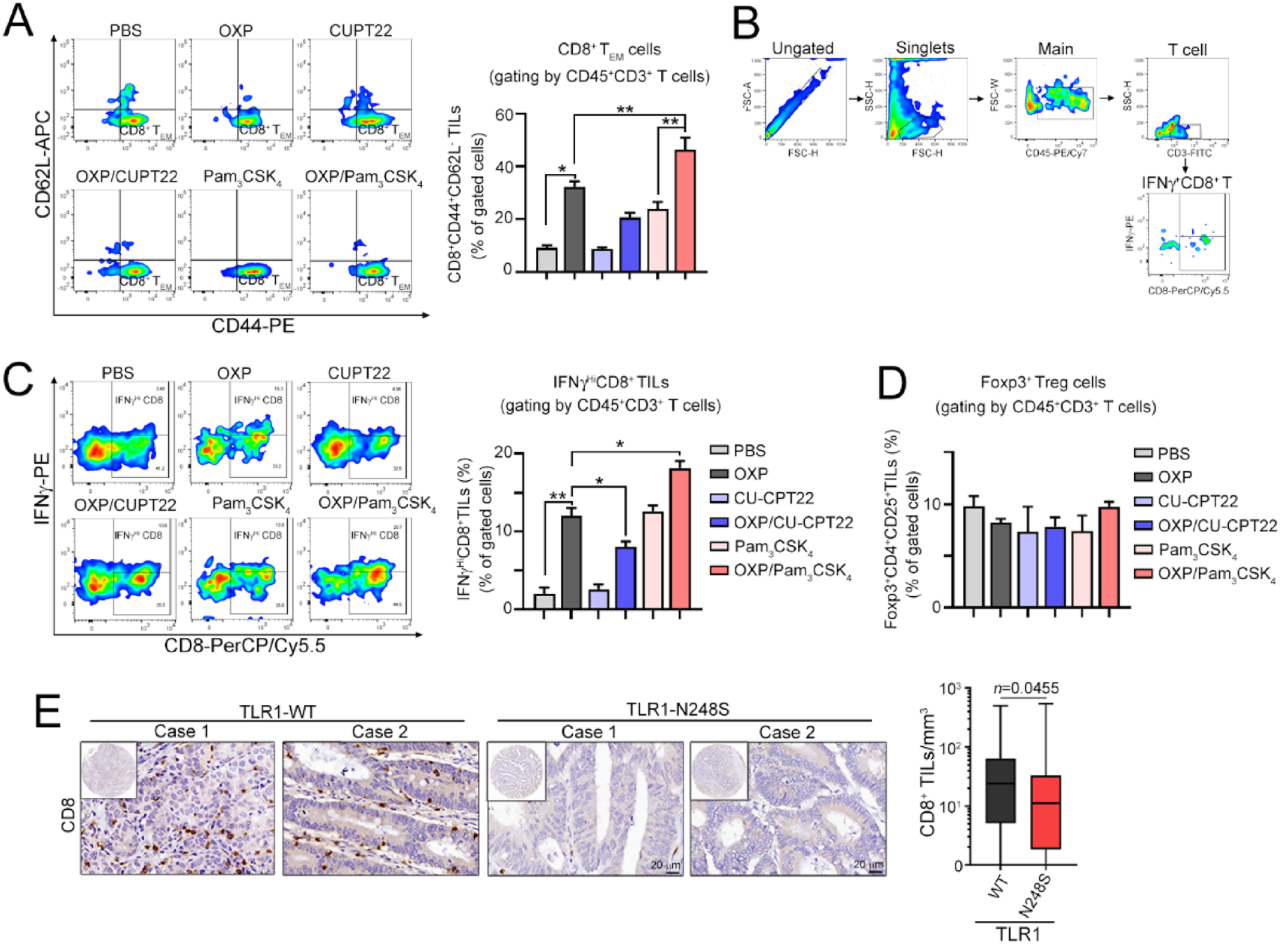
Inhibition of TLR1/2 signaling remarkably impacts the infiltration of immune cells after immunogenic chemotherapy treatment in vivo. (**A**) The effector/memory cells of CD3^+^CD45^+^ tumor-infiltrating immune cells were analyzed by flow cytometry (*n* = 4–5). The percentage of CD8^+^ T_EM_ cells (CD44^+^CD62L^-^CD8^+^CD3^+^CD45^+^ T cells) in total T cells (CD3^+^CD45^+^ T cells) was examined. One-way ANOVA t test, *p < 0.05 and **p < 0.01. (**B**) Gating strategy of the IFNγ^+^CD8^+^ tumor-infiltrating immune cell profile. (**C**) IFNγ^+^CD8^+^ tumor-infiltrating immune cells were analyzed by flow cytometry (*n* = 4–5). The percentage of IFNγ^+^CD8^+^ T cells (IFNγ^+^CD8^+^CD3^+^CD45^+^ T cells) in total T cells (CD3^+^CD45^+^ T cells) was examined. One-way ANOVA t test, *p < 0.05 and **p < 0.01. (**D**) The Foxp3^+^ T regulatory cells were analyzed by flow cytometry (*n* = 4–5). The percentage of Foxp3^+^Treg cells (Foxp3^+^CD4^+^CD25^+^ T cells) in total T cells (CD3^+^CD45^+^ T cells) was examined. One-way ANOVA t test. (**E**) The density of tumor-infiltrating CD8 cells within the tumor microenvironment in patients with locally advanced colon carcinoma was analyzed by immunohistochemistry. The association between CD8 density and TLR1-N248 polymorphism was analyzed (*n* = 410, *p* = 0.0455). Unpaired t test.

By evaluating the density of effector/memory CD8 cells, we found that there was a remarkable decrease in the OXP/CU-CPT22 group but an increase in the OXP/Pam_3_CSK_4_ group (Fig. 4A). The tumor-infiltrating IFNγ^+^ CD8 cells were also increased in the OXP/Pam_3_CSK_4_ group (Fig. 4B,C). However, we found no significant change in immunosuppressive Foxp3^+^ regulatory T cells within TME (Fig. 4D). Taken together, these results showed that OXP can induce infiltration of effector/memory and cytotoxic immune cells to recognize and eradicate residual tumor cells via TLR1/2-mediated signaling, indicating that TLR1/2 is critical to enhance the therapeutic efficacy of immunogenic chemotherapy. Additionally, the density of tumor-infiltrating CD8^+^ T cells was significantly lower in locally advanced stage III COAD patients bearing the TLR1-N248S polymorphism (Fig. 4E). Taken together, these results showed that defective TLR1 may reduce the therapeutic efficacy of chemotherapy in locally advanced COAD patients by attenuating HMGB1-mediated antitumor immunity.

## Discussion

Our data showed for the first time that TLR1-N248S was associated with the clinical outcome of patients with locally advanced stage III COAD, suggesting that this genetic defect might be associated with the risk of lymph node metastasis and poor DMFS. We found that defective TLR1 might attenuate HMGB1-mediated ICD to decrease the response to immunogenic chemotherapy, leading to a reduced antitumor response and poor therapeutic efficacy in vitro and in vivo. Furthermore, patients carrying the TLR1-N248S polymorphism had fewer tumor-infiltrating CD8^+^ lymphocytes within the TME in locally advanced COAD and associated with poor DFS. These findings indicate that TLR1 signaling plays a critical role in the therapeutic efficacy of chemotherapy by modulating antitumor immunity.

Our previous studies reported that the extent of HMGB1 release and immune cell infiltration were associated with favorable survival outcomes in locally advanced rectal cancer patients after preoperative chemoradiotherapy treatment^26^, suggesting that the HMGB1-TLR2/4 signaling pathway may participate in chemotherapy-induced antitumor immunity. TLR2 can form heterodimers with TLR1 or TLR6 to recognize different TLR ligands, resulting in different functions^46^. Previous studies showed that activation of TLR1/2 signaling could activate the immune response, but activation of TLR2/6 signaling could suppress T-cell immunity^47,48^. Moreover, recent studies have shown that TLR1/2 agonists enhance the antitumor efficacy of CTLA-4 and PD-L1 blockade by increasing intratumoral Treg depletion^12,49^ and stimulating cytotoxic T lymphocytes against tumor cells^11^. Here, our studies showed that TLR1 is essential for TLR1/2 signaling activation in DCs in response to immunogenic chemotherapy. We assumed that defective TLR1 may attenuate TLR1/2 signaling but enhance TLR2/6 signaling to suppress T-cell immunity. Therefore, HMGB1 released by immunogenic chemotherapy may activate TLR2/6 signaling in DCs to suppress T-cell-mediated immunity rather than promote the T-cell-mediated immune response in COAD patients with defective TLR1. Indeed, by silencing TLR1 expression on immature dendritic cells, we found that TLR2 signaling was less activated by HMGB1 compared to wild-type immature dendritic cells. Moreover, direct coculture of OXP-treated colorectal cancer cells with defective TLR1-iDCs showed that DC maturation and T-cell activation were remarkably attenuated in vitro, suggesting that TLR1 is essential for the HMGB1-TLR2 axis to boost antitumor immunity. Furthermore, systemic inhibition of TLR1/2 signaling markedly reduced the response to chemotherapy, leading to tumor regrowth. However, systemic administration of a TLR1/2 agonist significantly delayed tumor growth and increased the response to chemotherapy. Within the tumor microenvironment, we found that inhibition of TLR1/2 by the small molecule CU-CPT22 dramatically decreased the effect of OXP, leading to fewer tumor-infiltrating immune cells, including CD4^+^, CD8^+^ and IFNγ^+^ CD8^+^ cells. However, coadministration of a TLR1/2 agonist boosted the influence of immunogenic chemotherapy, increasing antitumor immunity to reshape the TME and eradicate residual tumors. Consistent with these observations, we found a lower density of tumor-infiltrating CD8^+^ lymphocytes within the tumor microenvironment in locally advanced colon carcinoma bearing the TLR1-N2418S polymorphism. Notably, we found no significant change in regulatory T (Treg) lymphocytes in these groups, which is inconsistent with previous studies^47^. Zhang et al*.* found that the TLR1/2 ligand not only promoted cytotoxic T lymphocyte infiltration but also reduced the infiltration of Tregs^47^. Sharma et al*.* also indicated that a TLR1/2 agonist enhanced the efficacy of an anti-CTLA-4 antibody by depleting regulatory T cells and increasing IFN-γ secretion from T cells to reshape the TME in a syngeneic melanoma model^12^. We assumed that the tumor immunophenotype might be different in these models. Our syngeneic colon model CT26 had more infiltration of Tregs than the melanoma Model B16F10^50,51^, which may influence the impact of TLR1/2 agonists on the immune status within the TME. These divergences need to be investigated in different syngeneic animal models in the future.

Previous reports have shown that several SNPs in TLR1 genes may modify the activation of its protein, implying that the genetic variation in the TLR1 gene might affect the innate immune response and clinical susceptibility to a wide spectrum of pathogens and confer a high risk of cancer^52^, including N248S and I602S. Here, we found that the TLR1-N248S polymorphism was not only associated with poor survival outcomes but also influenced the response to adjuvant chemotherapy. Consistent with our studies, Okazaki et al. also indicated that TLR1-I602S (rs5743618) could serve as a predictor of clinical response to FOLFIRI plus bevacizumab in patients with mCRC^15^. Moreover, Deng et al*.* showed that TLR1/2 expression had a positive correlation with lung cancer patient survival^46^, suggesting that TLR1/2 signaling may influence the therapeutic response to chemotherapy by modulating antitumor immunity. Therefore, low TLR1/2 expression or defective TLR1/2 signaling may result in poor clinical benefit of chemotherapy, especially immunogenic chemotherapeutic agents.

Taken together, our studies showed that the TLR1-N248S polymorphism is not only a prognostic but also a predictive factor for the clinical response to adjuvant chemotherapy in patients with locally advanced colon carcinoma. The TLR1-N248S polymorphism might reduce the ability of immunogenic chemotherapeutic agents to reshape the tumor microenvironment, leading to an insufficient antitumor immune response and tumor recurrence.

### Supplementary Information

Supplementary Information 1.

Supplementary Table.

## Data Availability

All datasets generated or analyzed during the current study are included in this published article.
