# Supplementary figures and images for: Dysfunctional TLR1 reduces the therapeutic efficacy of chemotherapy by attenuating HMGB1-mediated antitumor immunity in locally advanced colorectal cancer
Source: Sci Rep. 2023 Nov 9;13:19440. doi: 10.1038/s41598-023-46254-1 (PMC10636035; doi:10.1038/s41598-023-46254-1)

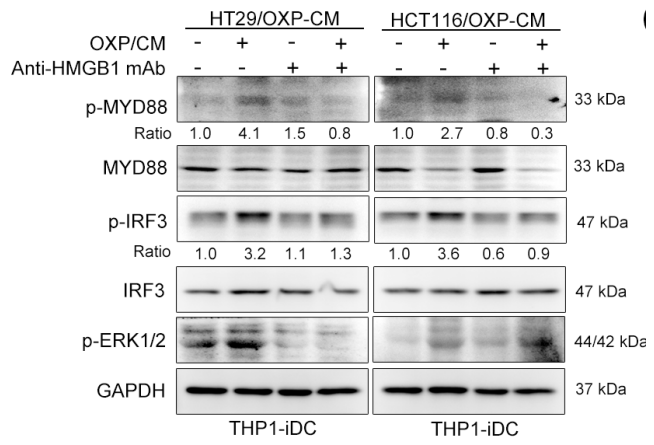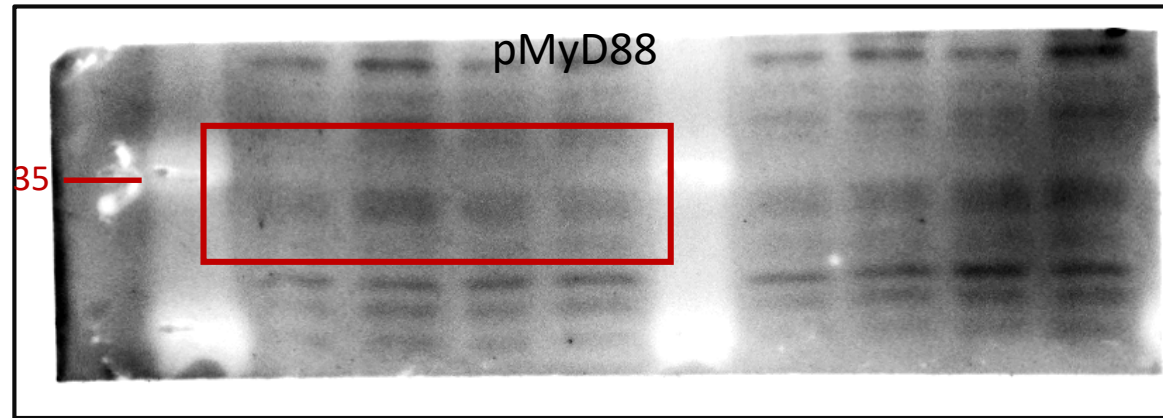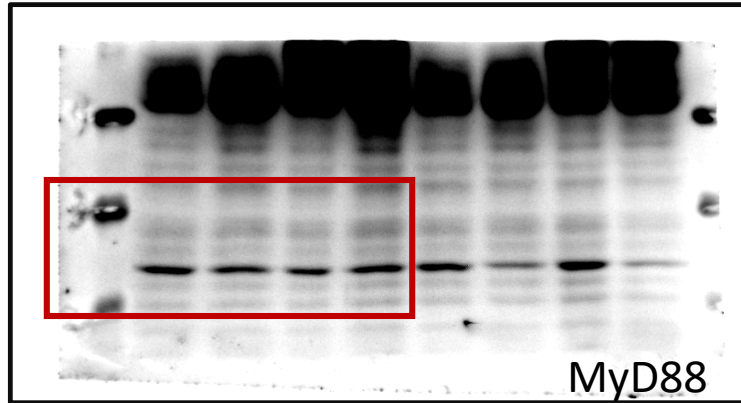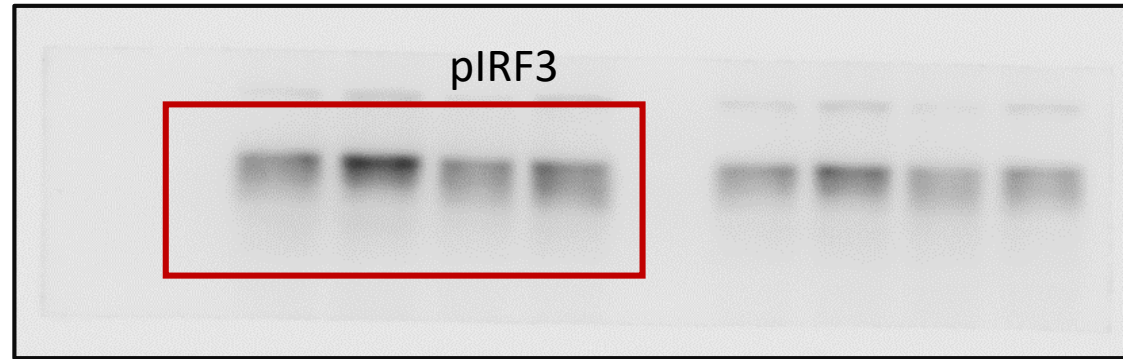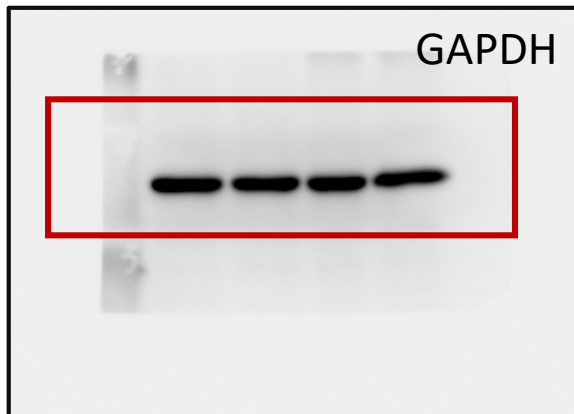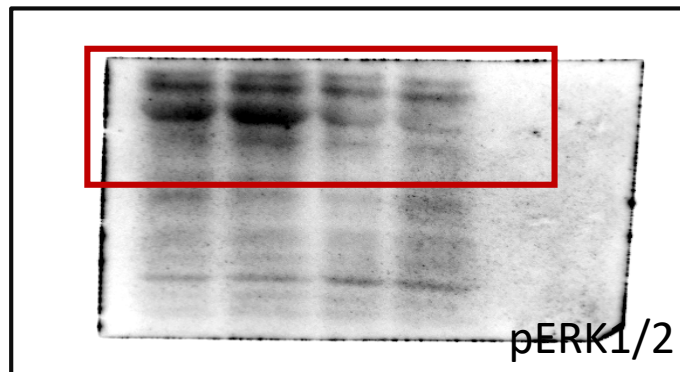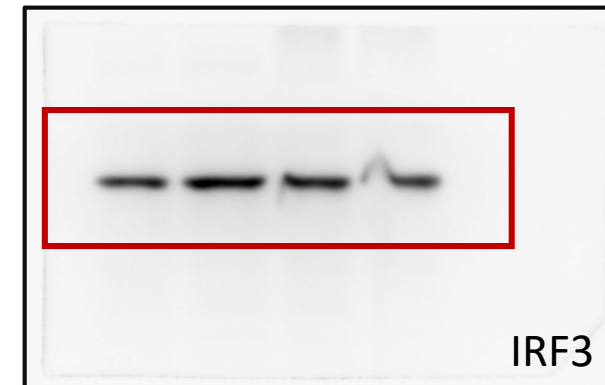

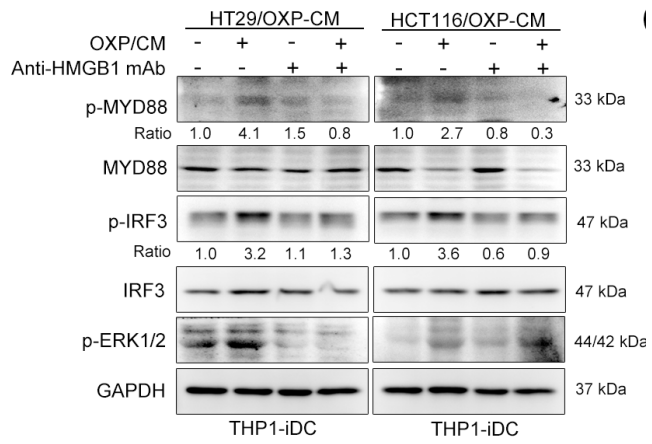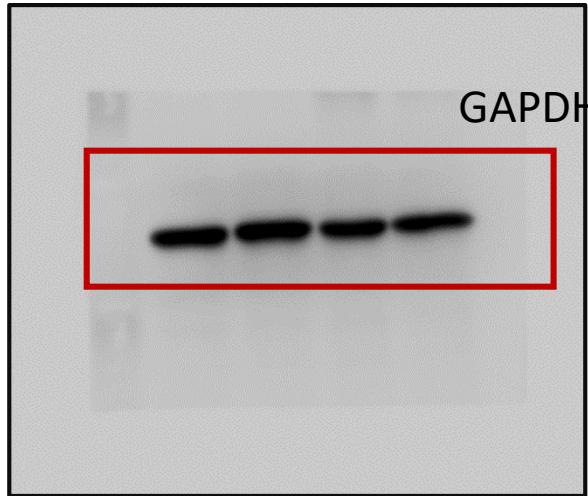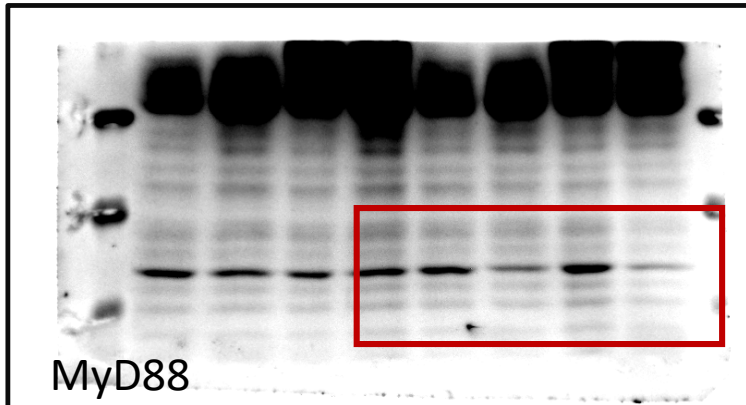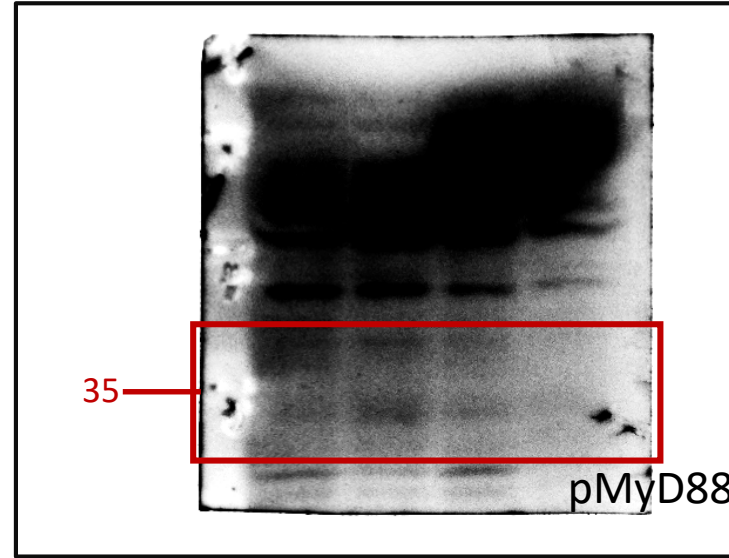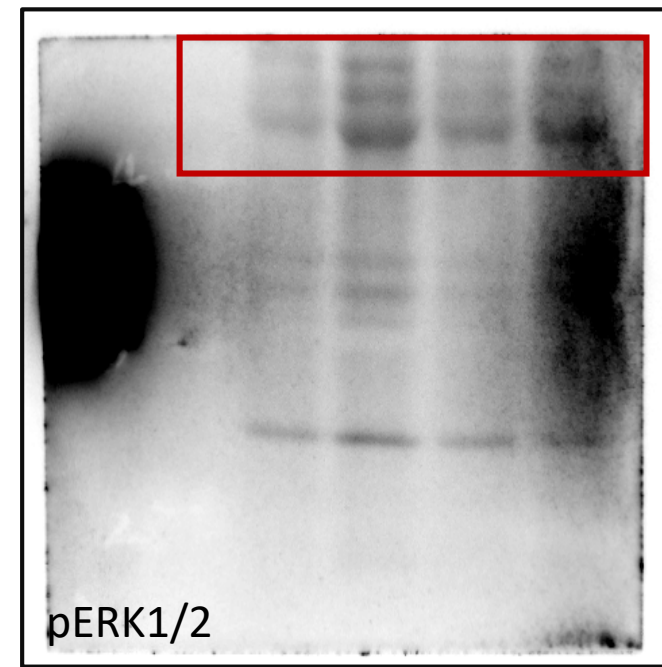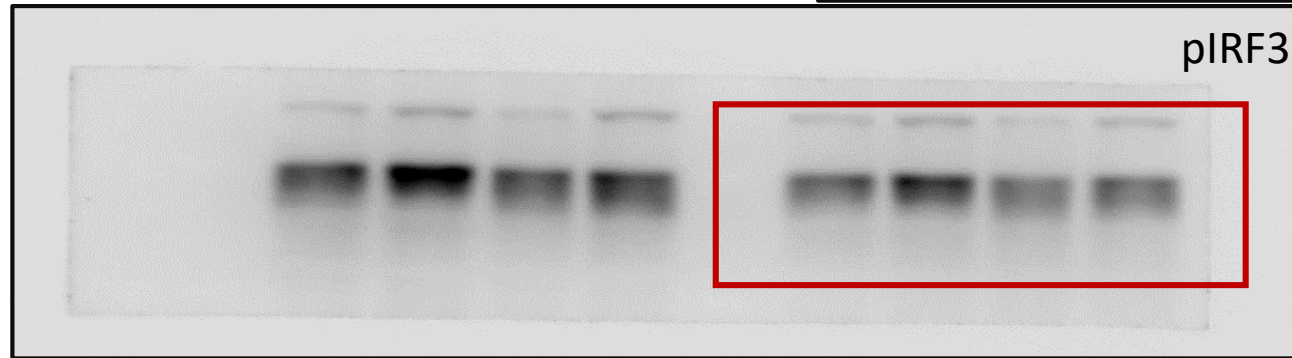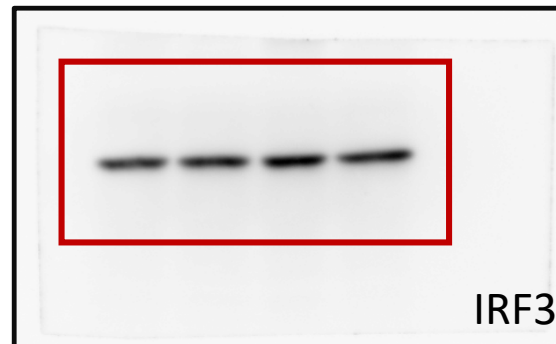

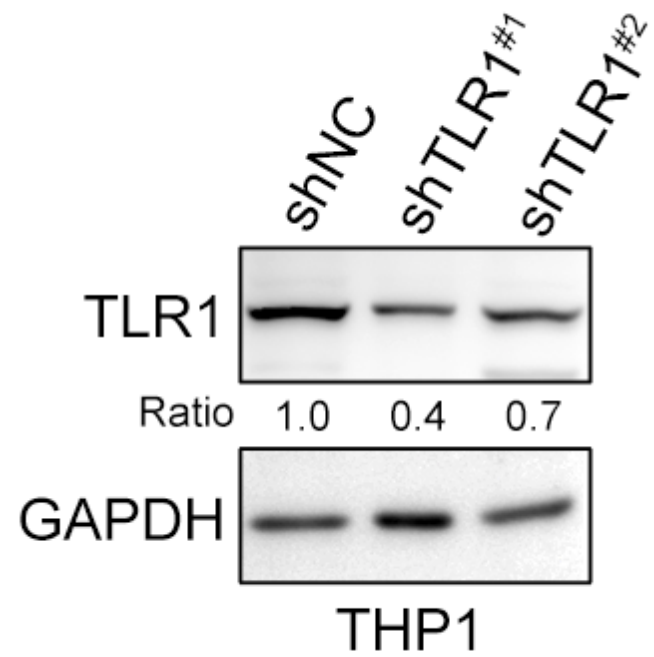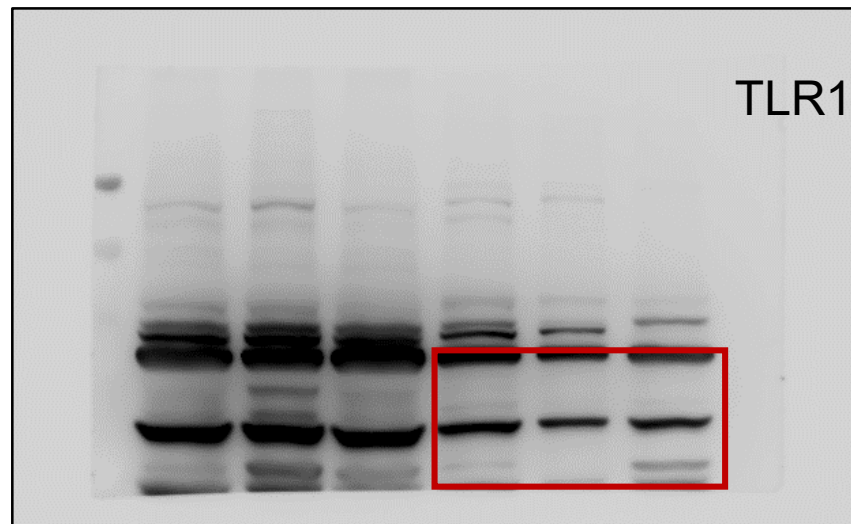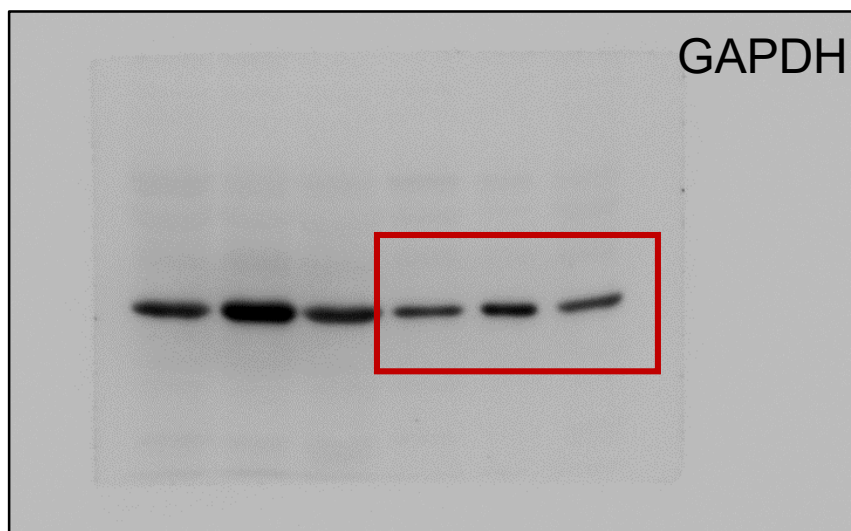

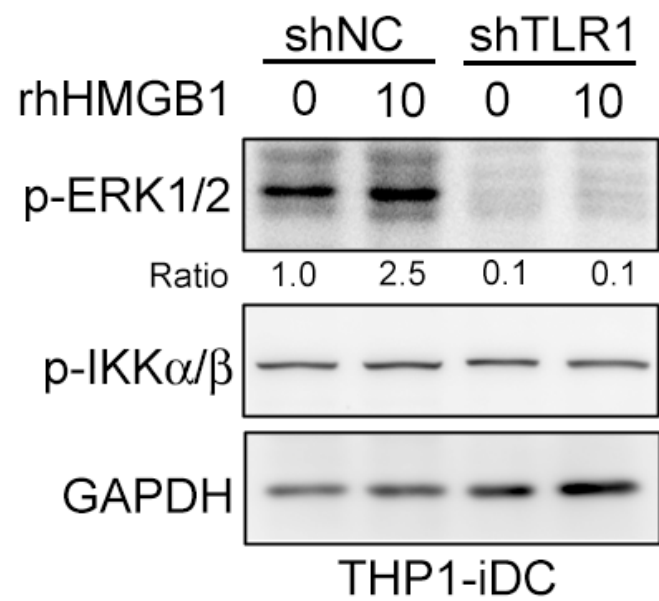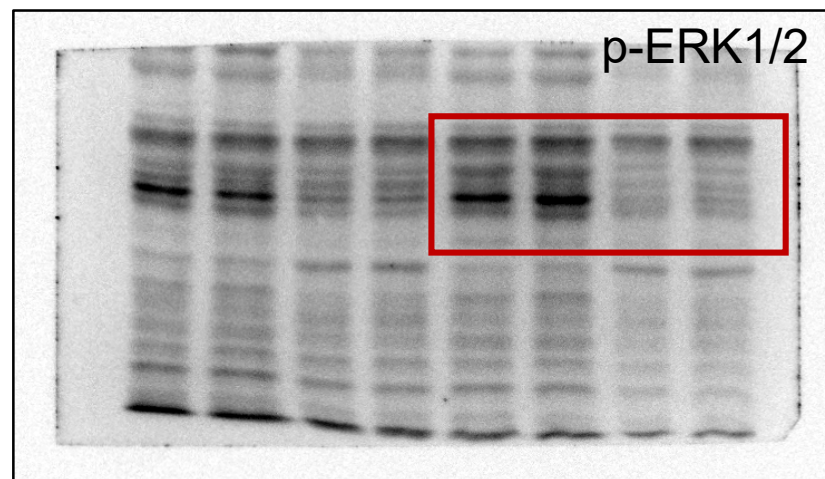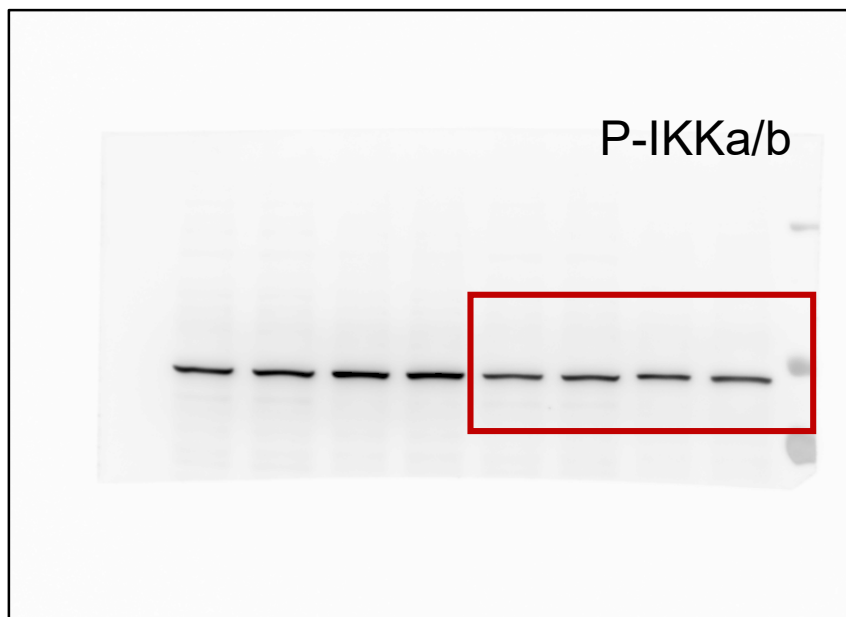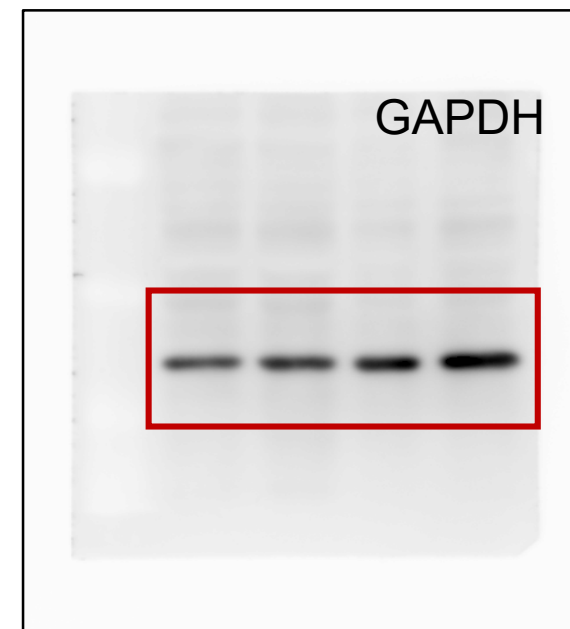

Supplement: Supplementary file 1 — Supplementary Information 1. [file 41598_2023_46254_MOESM1_ESM.pdf]
