# Supplementary material for: Dysfunctional TLR1 reduces the therapeutic efficacy of chemotherapy by attenuating HMGB1-mediated antitumor immunity in locally advanced colorectal cancer
Source: Sci Rep. 2023 Nov 9;13:19440. doi: 10.1038/s41598-023-46254-1 (PMC10636035; doi:10.1038/s41598-023-46254-1)
Supplement: Supplementary file 2 — Supplementary Table. [file 41598_2023_46254_MOESM2_ESM.docx]

| **Table S1. SNPs and Primers** | | | | | | |
| --- | --- | --- | --- | --- | --- | --- |
|  | **SNP** | **MAF^a^** | **Base change (AA change)** |  |  | **Primer sequence** |
|  | rs5743611 |  | C >G R80T | 1st |  | ACGTTGGATGCCAATTCCTGGTTGAATTTG |
|  |  | 0.0003 |  | 2nd |  | ACGTTGGATGTATCACTGTCAAAACTGAGG |
|  |  |  |  | UEP |  | AACACTGATATCAAGATACTGGATT |
| TLR1 | rs4833095 | 0.604 | A > G N248S | 1st |  | ACGTTGGATGCCTAAGTATTCTGGCGAAAC |
|  |  |  |  | 2nd |  | ACGTTGGATGCTGGAGGATCCTAATGAAAG |
|  |  |  |  | UEP |  | TCAATGTTGTTTAAGGTAAGA |
|  | rs5743618 | 0.006 | G > C S602I | 1st |  | ACGTTGGATGGCTGATCGTCACCATCGTTG |
|  |  |  |  | 2nd |  | ACGTTGGATGTGAGATACCAGGGCAGATCC |
|  |  |  |  | UEP |  | GGGCAGATCCAAGTAG |
| TLR2 | rs61735278 | 0.001 | A >G N729S | 1st |  | ACGTTGGATGGTGGTGCAAGTATGAACTGG |
|  |  |  |  | 2nd |  | ACGTTGGATGCAATGGGCTCCAGAAGAATG |
|  |  |  |  | UEP |  | gagagTGAGAATGGCAGCATCA |
|  | rs111200466 | 0.352 | Deletion (-196 to -174 deletion) | 1st |  | ACGTTGGATGCCTTTTGCTTACTTCCTAGTC |
|  |  |  |  | 2nd |  | ACGTTGGATGGGTGCAGAGAGACCACACG |
|  |  |  |  | UEP |  | CCGAGCAGTCACCTG |
| TLR4 | rs4986790 | 0 | A >G D299G | 1st |  | ACGTTGGATGAGCATACTTAGACTACTACC |
|  |  |  |  | 2nd |  | ACGTTGGATGCACACTCACCAGGGAAAATG |
|  |  |  |  | UEP |  | GTCAAACAATTAAATAAGTCAATAATA |
|  | rs4986791 | 0 | C >T T399I | 1st |  | ACGTTGGATGAGCCCAAGAAGTTTGAACTC |
|  |  |  |  | 2nd |  | ACGTTGGATGAGGTTGCTGTTCTCAAAGTG |
|  |  |  |  | UEP |  | gCAAAGTGATTTTGGGACAA |
| ^a^Minor allele frequency (MAF), according to the ALFA allele frequency for Asian. | | | | | | |
